# Role of Pectoralis Major Myocutaneous Flap in Laryngectomy Surgery: Single Surgeon Experience

**DOI:** 10.7759/cureus.18198

**Published:** 2021-09-22

**Authors:** Samba Siva Bathula, Noah A Stern, Andrew Ross, Tyler Patrick, Edward R Talatala

**Affiliations:** 1 Otolaryngology, Michigan State University/Detroit Medical Center, Detroit, USA; 2 Otolaryngology, Detroit Medical Center, Detroit, USA; 3 Otolaryngology - Head and Neck Surgery, Detroit Medical Center, Detroit, USA; 4 Otolaryngology - Head and Neck Surgery, Meharry Medical College, Detroit, USA

**Keywords:** flap, regional flap, pharyngeal fistula, fistula, pcf, pectoralis major myocutaneous flap, pmmc flap, salvage laryngectomy, laryngeal cancer, pharyngocutaneous fistula

## Abstract

Objective

To analyze the effectiveness of the pectoralis major myocutaneous (PMMC) flap in preventing pharyngocutaneous fistula (PCF) formation for salvage total laryngectomy patients.

Study design

Retrospective chart review of all post-surgical laryngectomy patients for a single surgeon.

Methods

Inclusion criteria were adult patients 18 years and older who were diagnosed with laryngeal cancer and treated with a salvage total laryngectomy. Exclusion criteria were any laryngectomy patient treated without the PMMC flap or those with incomplete medical records.

Results

A total of 31 patient charts were identified, and 16 remained after exclusion criteria. The patient age range was 42-71 years (mean = 58.19; SD = 8.093). Greater than 85% of patients had T3 and T4 laryngeal cancers. Without PCF group were 13 patients and with PCF group were only three patients. The mean hospital days in without PCF group were 9.54 and in with PCF group were 16.33.

Conclusion

In this single surgeon’s experience, PCF was prevented by using the PMMC flap in salvage total laryngectomy patients.

## Introduction

Squamous cell carcinoma of the larynx is the most common type of malignant lesion in the larynx and is most often treated with organ preservation chemoradiation therapy according to the new guidelines [1,2]. Total laryngectomy is usually indicated for advanced laryngeal cancers, such as T4 laryngeal cancer. It is also indicated for persistent and recurrent disease after chemoradiation therapy [3,4]. Pharyngocutaneous fistula (PCF) is more often seen in salvage laryngectomy than with primary laryngectomy due to radiation damage to the microvascular structure, causing delayed wound healing [5,6]. Several different types of techniques and methods have been used to prevent PCF such as pectoralis major myocutaneous (PMMC) flap and pedicle free flaps [7,8].

Our null hypothesis is that there is no significant utility of PMMC flap to prevent and manage PCF formation in salvage total laryngectomy.

## Materials and methods

This investigation employed a retrospective research methodology. Data was gathered from our existing clinical-surgical database at Detroit Medical Center between January 1, 2013 and December 31, 2019. We examined in detail both in-patient and out-patient clinic charts for information related to patient demographics and clinical information was collected.

Any patient diagnosed with laryngeal cancer with laryngectomy over the age of 18 years was included. Any laryngectomy patient who was not treated with PMMC flap and incomplete medical records was excluded.

Statistical analyses

SPSS (IBM Inc., Armonk, USA) power analysis with multinomial regression at 0.8 set power with seven total predictors suggested that at least 12 subjects will be needed for analytical purposes. SPSS Statistics were used for descriptive statistical analysis, including chi-square and Fisher's exact test calculations for comparisons among the categorical variables. Multinomial regression analysis was performed to assess the effectiveness of PMMC flap in laryngectomy patients to prevent PCF with the statistical significance of p < 0.05.

## Results

PMMC flap technique: The surface markings of the PMMC flap were made by drawing a line from the left (author preferred left side for right dominant hand patients) acromion to the xiphisternum. Another line vertically from the midpoint of the clavicle intersects the first line. The pectoral branch of the thoracoacromial artery usually runs along the vertical line.

**Figure 1:**
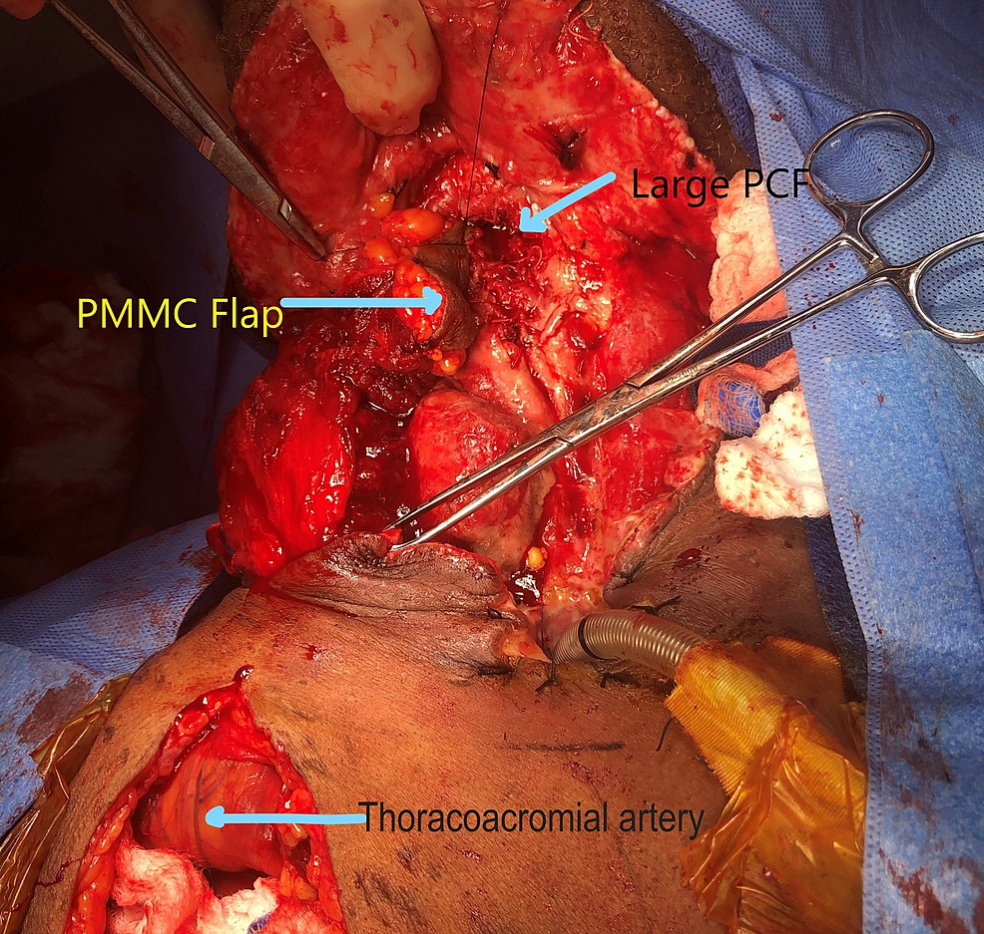
Thoracoacromial artery, large PCF, and PMMC flap. PMMC: Pectoralis major myocutaneous; PCF: Pharyngocutaneous fistula.

Size and location of skin paddle were dependent on the defect size and usually in the inferior border. The initial skin incision was at the design of the skin paddle and carried down deep to the pectoralis major muscle. The skin paddle was secured with to pectoralis muscle with 3-0 Vicryl to prevent any avulsion of skin paddle from pectoralis muscle during flap elevation. Then skin incision was extended along the vertical line superiorly up to the inferior border of the clavicle and the skin flap was separated from the pectoralis major muscle facia. The inferior border of the pectoralis muscle was identified and was separated from the chest wall along with its deep facia. Medial and lateral incision were made to identify thoracoacromial vessels. PMMC flap was elevated from chest wall inferior to superior direction without disrupting the thoracoacromial vessels with a cold knife and bipolar cautery. Author always keep adequate amount of facia over the thoracoacromial vessels to prevent vasospasm after flap elevation as shown in Figure 1.

**Figure 2:**
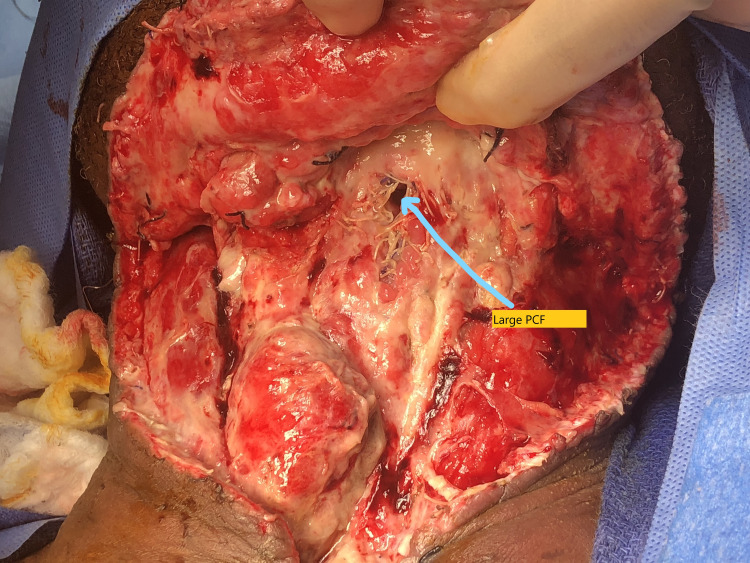
Large PCF. PCF: Pharyngocutaneous fistula.

**Figure 3:**
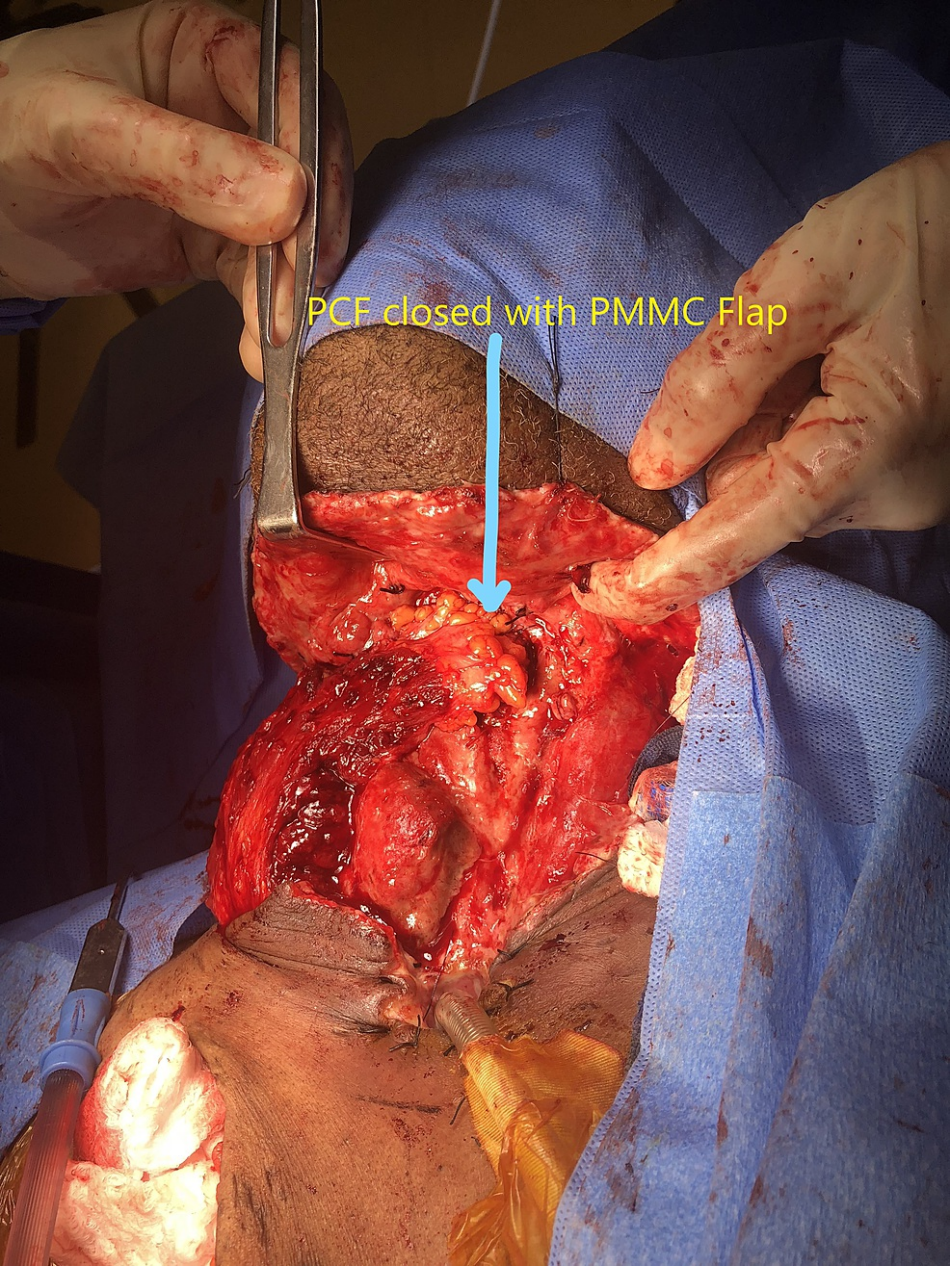
Large PCF closed with PMMC flap. PMMC: Pectoralis major myocutaneous; PCF: Pharyngocutaneous fistula.

The lateral thoracic artery was also saved most often. Any chest wall perforators encounter during flap elevation were ligated. Then the subcuticular tunnel was created over the clavicle from the chest wall into the neck-PMMC flap pulled into the neck without tension. PMMC flap was usually placed immediately over the neopharynx. If any defect was encountered during neopharynx closure or large PCF, PMMC flap was used to close the defect, as shown in Figures 2-3.

The examined dependent variables were PCF formation and postoperative hospitalized days. The independent variables were total salvage laryngectomy with PMMC flap first (no PCF) and total salvage laryngectomy with PCF managed by PMMC flap (PCF). Additionally, two covariates were gender and age.

**Figure 4:**
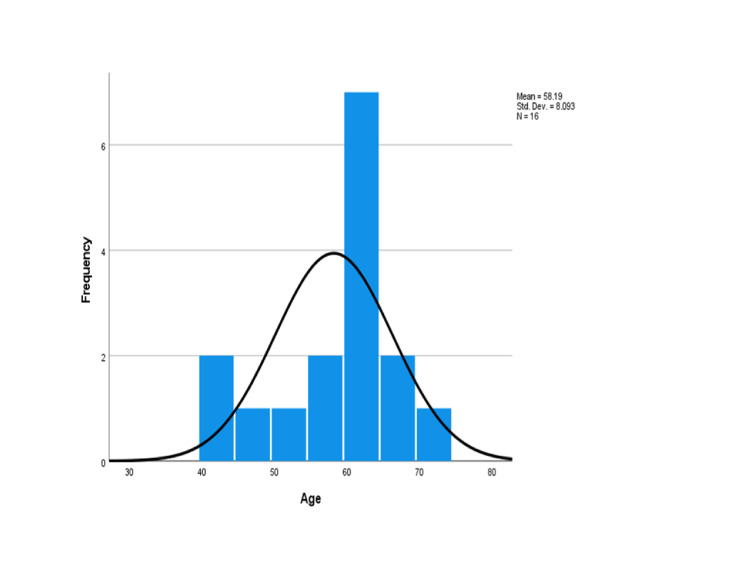
Histogram illustrating age distribution of patients included in this study.

A total of 31 patient charts were reviewed and only 16 met the inclusion and exclusion criteria. Only 12% (n = 2) were females. The age range was 42-71 years (mean = 58.19; SD = 8.093; Figure 4).

More than 85% patients had T3 and T4 laryngeal cancers (Table 1).

**Table 1:** Cancer initial clinical staging of patients included in this study.

Cancer initial clinical staging	Frequency	Percent
T1b N0 M0	1	6.3
T2 N0 M0	1	6.3
T3 N0 M0	5	31.3
T3 N2b M0	1	6.3
T4a N0 M0	1	6.3
T4a N1 M0.	1	6.3
T4a N2b M0	3	18.8
T4a N2c M0	3	18.8
Total	16	100

Multivariate regression analysis of patient demographics with salvage laryngectomy shows no significance for variables, including age (χ2 = 0.225, p = 0.635), sex (χ2 = 0.0002, p = 0.987), hypertension (χ2 = 0.857, p = 0.355), or diabetes mellitus (χ2 = 0.04, p = 0.841) except number of hospitalized days (χ2 = 3.8, p =0.051; Table 2).

**Table 2:** Salvage laryngectomy relationship among age, sex, hypertension, diabetes mellitus, and number of hospital days.

	Chi-square	Sig.
Age	0.225	0.635
Sex code = male-1, female-0	0.0002	0.987
Hypertension	0.857	0.355
Diabetes mellitus	0.04	0.841
Hospitalized days	3.8	0.051

The mean hospital days in without fistula formation group were 9.54 and in with fistula formation group were 16.33 (Table 3).

**Table 3:** Hospitalized days for PMMC flap along with total laryngectomy (no PCF) versus PMMC flap after fistula formation (PCF). PMMC: Pectoralis major myocutaneous; PCF: Pharyngocutaneous fistula.

Salvage total laryngectomy	PMMC flap along with total laryngectomy (no PCF)	PMMC flap use after fistula (PCF)
N	13	3
Mean	9.54	16.33
SD	1.391	1.528
Hospitalized days-Minimum	9	15
Hospitalized days-Maximum	14	18

## Discussion

Salvage total laryngectomy carries significant postoperative complications, and PCF (around 30% without vascularized flap) is the most concerning complication associated with significant morbidity and mortality [9].

Radiation induces microvascular injury leading to extensive scarring, fibrotic tissue remodeling, and altered perfusion of irradiated tissue [10]. Inadequately perfused tissue always carries poor wound healing. This is the most common reason PCFs are usually seen within the first 10 postoperative days in salvage laryngectomies. Various vascularized tissue flaps, such as free flaps and locoregional flaps have been used to prevent PCF [11]. The exact mechanism of action of vascularized flaps was not known. Vascularized tissue flaps have abundant hyaluronic acid in the deep muscular fascia, which may be an extra promoter of healing [12].

PMMC flap was first described by Ariyan S in 1979 and was widely popular in salvage laryngectomy patients to prevent PCF [13]. Fung K et al. reported free vascularized flaps were effective in preventing major wound complications but did not reduce the overall fistula rate [14]. Higgins KM et al. used temporoparietal fascial flap (TPFF) in salvage laryngectomy patients, and their PCF rates were similar to those with pectoralis major muscle flaps [15].

Gabrysz-Forget F et al. reported free flaps were associated with a longer operative time, a higher cost, and a higher incidence of postoperative revisions compared to pedicle flaps [12]. PMMC flaps are favorable in salvage laryngectomies for these reasons.

In our study, only one patient developed a fistula after total laryngectomy with PMMC flap due to prior bilateral chest radiation therapy for breast cancer. Iglesias-Moreno MC et al. also reported similar PCF rates in salvage laryngectomy with PMMC flap [16]. We had three patients with large PCF after salvage total laryngectomy, most probably due to radiation-induced microvascular injury leading to poor wound healing. All three were managed with PMMC flap within 24 hours after fistula diagnosis due to a rich vascularized flap with abundant hyaluronic acid in its deep muscular fascia.

Each patient underwent a tracheoesophageal puncture and had a reliable voice. The mean hospitalized days were significantly less in total laryngectomy in PMMC flap patients and are consistent with available literature [17]. The only disadvantage of PMMC flap was the restriction of arm adduction, apart from aesthetic considerations.

Since we have only a small number of subjects, we cannot make a clear recommendation. However, we found it is valuable to share our results at the time of widespread use of free flaps in salvage laryngectomy patients.

## Conclusions

Squamous cell carcinoma is one of the most common cancers in the larynx. Total laryngectomy is a common treatment modality for advanced T4 laryngeal cancers and persistent and recurrent laryngeal disease after chemoradiation therapy.

One of the most concerning complications of salvage total laryngectomy is a PCF. PMMC flap is a reliable surgical treatment option for the prevention and management of large PCF.
